# Distribution and symmetrical patellofemoral pain patterns as revealed by high-resolution 3D body mapping: a cross-sectional study

**DOI:** 10.1186/s12891-017-1521-5

**Published:** 2017-04-18

**Authors:** S. A. Boudreau, E. N. Kamavuako, M. S. Rathleff

**Affiliations:** 1 Department of Health Science and Technology, CNAP, SMI® Aalborg University, Fredrik Bajers Vej 7, Aalborg, 9000 Denmark; 2 Research Unit for General Practice in Aalborg, Department of Clinical Medicine, Aalborg University, Aalborg, Denmark

**Keywords:** Digital pain drawings, Pain distribution, Patellofemoral pain, Chronic knee pain, Symmetry, Fuzzy logic, Adolescents, Young adults

## Abstract

**Background:**

Detailed pain mapping of extent and distribution in individuals with patellofemoral pain (PFP) within and around a complex structure such as the knee has yet to be explored.

**Methods:**

Perceptions of on-going pain from adolescents and young adults (*N* = 35) with long-standing (>10 months) PFP were collected on high-resolution 3D digital body-schema of the knees. Location, area of pain, pain intensity, laterality, worse side of knee pain, symptom duration, and symmetry in bilateral knee pain were recorded. A threshold for naturally occurring variations in symmetrical knee pain drawings were collected from 18 healthy controls and used in combination with the development a symmetry index (0–1) to create a fuzzy rule for classifying symmetrical and non-symmetrical PFP patterns as compared to a PFP expert. The symmetry index was computed and tested using a correlation coefficient alone or in combination with the Jaccard index and the true and false positive rates (TPR and FPR, respectively) determined.

**Results:**

The peripatellar region was the common report of pain location however, novel and nonconforming PFP patterns were identified and the majority of individuals (22 of 27) with bilateral PFP expressed highly-symmetric mirror-image pain. Individuals with symptom duration of 5 years or more had a greater area of pain, compared to those with symptoms for less than 5 years. The total area of pain was correlated to symptom duration for those with extended symptoms durations and a progression towards an “O” shaped pattern emerged. A TPR of 100% for identifying symmetrical knee pain patterns was found however the expert PFP tended to be stricter, as reflected in FPR of 20%.

**Conclusions:**

A high proportion of PFP patterns or symptoms occur in mirrored locations and are exceptionally symmetrical, and long duration of symptoms appear to converge to an ‘O’ shape. Classifying symmetrical pain patterns is subjective however simple fuzzy rules and correlations can be used to increase objectivity. This study highlights a gap in knowledge of PFP symptom presentation, reveals what may be a natural progression of symptoms, and provides valuable clinical insight for both pain management and treatment.

**Electronic supplementary material:**

The online version of this article (doi:10.1186/s12891-017-1521-5) contains supplementary material, which is available to authorized users.

## Background

Knee pain is highly common complaint amongst the general population, especially among adolescents [1] and more than 50% will at some point contact their general practitioner to seek treatment [2–4]. One of the most troublesome conditions among adolescents with knee pain is patellofemoral pain (PFP) affecting 6–7% [5–7]. Adolescent PFP is commonly considered a benign knee pain condition with good long-term prognosis, but recent evidence suggests the majority of adolescents with PFP experience knee pain for many years [8, 9]. A large proportion of these young individuals with PFP can expect to live the majority of their lives with knee pain and may have an increased risk of developing patellofemoral osteoarthritis [10–12]. The mechanisms (e.g. overuse or abnormal patellofemoral joint mechanics) and underlying pathology driving PFP remains unclear, however nociception could stem from several anatomical structures.

Traditionally, PFP is defined as retropatellar (behind the patella) or peripatellar (around the patella) knee pain. However, these may be crude divisions since the medial and lateral retinaculum, the patellar subchondral bone, the synovium, and the infrapatellar fat pad are all capable of producing anterior knee pain [13] Patients often describe diffuse anterior knee pain during activities that load the patellofemoral joint such as stair walking, walking or running. Individuals with PFP may communicate their pain by ‘placing both hands over their knees to indicate the area of pain’ or ‘point to the area around the knee such that pain is described as taking a classic or signiature *C-sign*’ [14]. Enabling patients to report or communicate their pain more clearly can be accomplished using three dimensional (3D) body-schemas [15] to indicate area and pain location more precisely, as traditional 2D line drawings provide little anatomical guidance for expression around complex structures, such as the knee. Furthermore, patients generally favoured the 3D body-schemas as compared to traditional line drawings (2D representations) of the body for reporting pain [15] and localized, regional, or diffuse pain has been reliability assessed on an artistic rendition of the knees, otherwise known as the Knee Pain Map [16]. Furthermore, conscious neurosensory mapping of the internal knee structure has shown that the anterior synovial tissues, fat pad, and capsule are extremely sensitive to mechanical stimuli and pain localization is perceived more accurately as compared to cruciate ligaments and menisci [17].

Recently, a positron emission tomography/computed tomography imaging study revealed that 44–85% of adults with PFP showed an increased tracer uptake (a proxy of tissue metabolic activity) in the patellofemoral joint that overlaps with areas of physiologic remodelling, such as bone marrow edema, subchondral bone cysts, and cartilage damage [18–20]. Furthermore, an association between tracer uptake and pain intensity as well as uptake location and pain location was found, suggesting an aetiological link [19]. Therefore, a more detailed account of pain location or pattern of referred pain could assist the identification of anatomical structures and underlying mechanisms contributing to PFP.

The aim of this study was to acquire detailed pain drawings of PFP patterns in adolescents and young adults and to investigate with more accuracy commonly referred pain locations and the associated area. This study also explored the development and initial testing of a symmetry index in response to the high proportion of symmetrical pain patterns found within adolescents and young adults with long-standing PFP. The symmetry index was combined with Fuzzy rules to incorporate a simple rule-based IF X AND Y THEN Z approach to classify pain drawings as symmetric or non-symmetric.

## Methods

### Subjects

This cross-sectional study included 14 healthy participants (eight females, age range 18–25 years) from the university environment were assessed for drawing accuracy in a symmetry drawing task and 36 individuals with PFP recruited from two separate on-going cohorts in order to get a wide range of symptom duration.

Cohort 1 consists of 16 adolescents (2 male) between 15 and 19 years of age recruited in the autumn of 2014. Cohort 2 consisted of 20 females between 18 and 22 years of age. They were randomly sampled from a larger cohort of 153 adolescents who has been followed for 3 years and was recruited in autumn 2011 when they were between 15 and 19 years of age. Both cohorts were initiated using a population-based recruitment procedure that has previously been described in detail [1]. In short, adolescents in upper secondary schools were asked to complete an online questionnaire on current musculoskeletal pain. If they reported knee pain they were called by telephone and a short anamnesis (case history) was collected. If they reported anterior knee pain with an insidious onset for more than 6 weeks, they were invited to a clinical examination. During the clinical examination the following inclusion criteria for PFP were used: insidious onset of anterior knee or retropatellar pain of more than 6 weeks duration and provoked by at least two of the following daily activities: prolonged sitting or kneeling, squatting, running, hopping, or stair walking; tenderness on palpation of the patella, pain when stepping down or double leg squatting; and worst pain during the previous week of more than 3 cm on a 10 cm visual analogue scale (VAS). Exclusion criteria were concomitant injury or pain from the hip, lumbar spine, or other knee structures; previous knee surgery; self-reported patellofemoral instability; knee joint effusion [9].

### Collection of self-reported outcomes

The following clinical self-reported measures that were used consisted of: 1) current pain intensity on a 0–10 numeric rating scale (NRS), 2) symptom duration (months), 3) most painful knee (right/left), 4) uni- or bilateral pain (yes/no). Cohort 1 completed all self-report outcomes at the time of inclusion in the autumn of 2014. Cohort 2 completed the same self-report outcomes during the same time period when they were seen at the local hospital for a 3 year follow-up. During the follow-up it was confirmed that they still suffered from PFP using the same criteria as when they were initially recruited for the cohort.

### Assessments of pain area and localisation

Individuals with PFP drew the area and location of the pain they experienced on a high resolution 3D body schema representing the leg and knees on a personal computer tablet (Samsung Galaxy note 10.1, 2014 Edition) using the Navigate Pain app (Aalborg University, Denmark) [15, 21]. For this study, the body schema represented the leg and knees such that the shadows in the 3D model displayed a clear delineation between patella and surrounding structures. The 3D image itself did not rotate and the perspective remained constant during data collection. Participants used an S-pen, which functions like a pen on paper, to further enable detailed drawings on the touch-screen interface. A single dot produced by the S-pen, in this study, marks approx. 8–10 pixels, using the default pen settings in the Navigate Pain app (Aalborg University, Denmark). Pain areas drawn with the touch-screen interface and S-pen in combination have a high-level of agreement and can be considered comparable to pain areas drawn with traditional pen and paper [15].

The area of pain associated with the left and right knee were individually extracted and expressed as total number of pixels using Navigate Pain. Each pain drawing was then analysed for pain location. The distribution of pain for each pain drawing was visually classified, independent of any other patient information, as clearly presenting with pain in (1) peripatellar only region, (2) retro only region or (3) a combination of both retro and peripatellar pain (mixed). Evidence of symmetry between the pain drawings of the left and right knee emerged and therefore the number of individuals expressing symmetrical or asymmetrical knee pain patterns was recorded and classified for further analysis. Expert assessments of symmetry considered shape and location of pain areas with respect to the patella, approximate location to the patellar tendon, fat pad, and ligaments. Thus, pain reported in the muscles was not used for classification. Individual knee pain drawings were merged as an overlay and plotted as heat grids using a custom Matlab® script in order to extract and visualise emerging patterns, common pain locations, and associated distributions.

### Symmetry drawing task and assessment

In response to high number of mirror image or symmetrical knee pain drawings found within individuals with PFP, a symmetry drawing task was designed to investigate the degree of accuracy that could be readily achieved if individuals intended to mirror their knee pain or intentionally create a symmetrical knee pain pattern. The goal of the symmetry drawing task was to provide data to develop an objective measure of the degree of symmetry (range 0 (no symmetry) – 1(perfect symmetry)) between two knee pain drawings that also accounts for human variability.

**Fig. 1:**
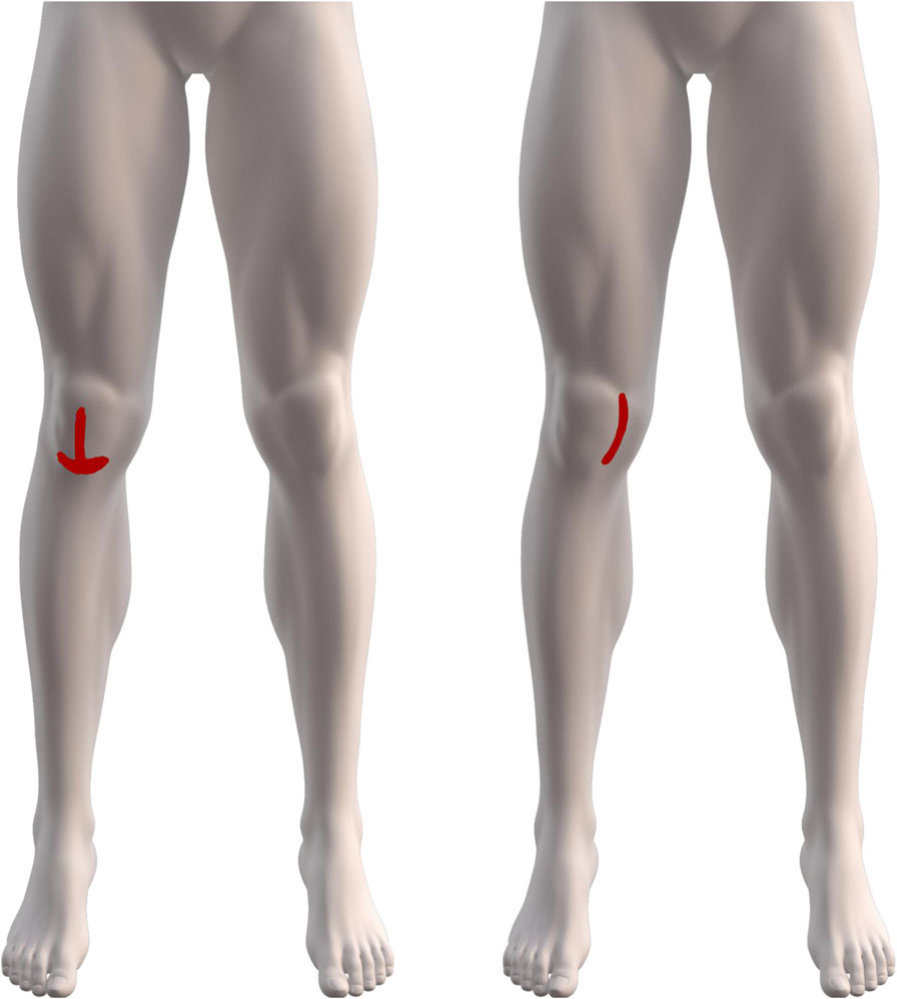
Knee pain drawings, redrawn in similarity to knee pain drawings acquired from the PFP cohort used the symmetry drawing task to assess natural variation

Two separate knee pain drawings, redrawn in similarity to knee pain drawings acquired from the PFP cohort, as shown in Fig. 1, were displayed in random order on a sheet of paper to each healthy participant. Healthy participants were instructed to draw the knee pain pattern displayed on the sheet of paper, onto one knee of the 3D body schema of the legs and knees. The sheet displaying the knee pain drawing was then covered and the healthy participant was then asked to mirror, to the best of their ability, the knee pain pattern they had just drawn onto the contralateral knee. The digital recordings of the knee pain drawings were saved and then classified by a PFP expert as symmetrical or non-symmetrical in order to develop an objective approach to assess symmetry.

### Objective assessment of symmetry

Among techniques for quantifying similarity between two images where the drawn area is either present or not present (binary) the correlation coefficient (CORR) and Jaccard index are appropriate [22]. For each symmetry drawing, the first knee pain drawing was flipped and then superimposed on the contralateral knee pain drawing in order to compute the two-dimensional (2-D) correlation coefficient (CORR1). The CORR1 represents the degree of similarity but it can be easily affected by possible differences in the location of the drawn areas. For example, the CORR1 would approach zero if the pain areas were located in two very distinct anatomical locations that may be perceived visually as same location. Given that there may be a minor difference in the location of the mirrored drawing and given that the goal was to assess the degree of symmetry, an additional assessment was computed which essentially translated the flipped drawing to maximise the overlap with the drawing on the contralateral knee. The translation coordinates reflecting the maximum overlap was identified by computing the maximum correlation coefficient (maxCORR) and from this location, the Jaccard index was computed. The Jaccard index reflects the degree of similarity or in this case, symmetry, between the two images and is defined as the size of the overlap divided by the total size or sum of the two pain drawings. Computation of the correlation coefficient takes into account the number of pixels where both images lack drawing (equal zero in binary), thus the size of surrounding zero pixels may affect this metric significantly. Jaccard index on the other hand does not account for those pixels and thus ensures consistency when determining the degree of symmetry. The drawings ascertained in the symmetry drawing task and the PFP data collection was then assessed using the CORR1 and also CORR2, here defined as (maxCORR + Jaccard index) divided by 2 to satisfy the degree of similarity ranging between 0 and 1.

The classifications as provided by the PFP expert were then used to develop a mathematical expression using the simple fuzzy rule IF X AND Y THEN Z, to distinguish symmetrical and non-symmetrical PFP patterns, where X = CORR1, Y = CORR2, and Z = symmetric or non-symmetric. For example, if CORR1 exceeds 0.5, the drawing is classified as symmetric, however CORR1 alone it not always robust. Therefore CORR2 was developed and simultaneously assessed. If CORR2 exceeds 0.5 the classification is symmetric.

### Statistical analysis

All calculations were performed using Sigma Stat 3.5 (Systat Software, California, USA). The cohort was analysed based on (1) symmetrical and asymmetrical knee pain patterns and (2) a dichotomized median split based on pain duration. Student’s *t*-test was used to assess differences in age, duration, pain intensity, and pain area as a result of the dichotomized median split in pain duration. Pain areas were then analysed between the left and right knee and side of worse pain using paired student t-tests or a two-way analysis of variance (ANOVA) with Group x left/right knee where relevant. The proportion of pain localisation for peripatellar only, retro only or mixed regions were analysed using a Fisher’s exact test. Similarly, the proportion of pain for lower and upper patellar regions were also analysed using a Fisher’s exact test. Spearman correlations were used to determine if there was a relationship between pain area and reported pain intensity. False positive rates (FPR) are expressed as the proportion of all non-symmetrical pain drawings (negatives) as specified by the PFP expert that yielded a symmetric (positive) classification by the fuzzy rule. Similarly, true positive rates (TPR) are expressed as the proportion of all symmetrical pain drawings as specified by the PFP expert that yielded a symmetric classification by the fuzzy rule. Means, standard deviations (SD) and 95% confidence intervals (CI) are reported where relevant. All data were normally distributed, unless expressed otherwise. P-values less than 0.05 were considered significant.

## Results

One patient drawing was excluded due to a meager (scribbled) record which did not appear to compromise assessment of pain location but rather the calculation of pain area. Therefore, patient demographics and knee pain characteristics of 35 individuals with long-standing PFP (range 10–162 months) are reported.

### PFP location

Only one patient presented with pain restricted to the retropatellar area whereas 57% presented with a pain pattern restricted in the peripatellar region, refer to Table 1. Less than half of the patients presented with a mixed pain pattern, that is, pain in both regions. In accord to a dichotomized median split of the 35 individuals based on symptom duration of 5 years (60 months) or more, there was no difference in the proportion of individuals presenting with peripatellar only or peripatellar and retropatellar pain (Fishers exact test, *p* = 0.357, Table 1).

**Table 1:** Knee pain area, location and associated demographics for the whole cohort and as a 5 year median split based on symptom duration (Less than 5 years vs. 5 years or more)

	Knee pain area (Pixels)			Pain location			Demographics		
	Total [95% CI]	Left [95% CI]	Right [95% CI]	Peri (n)	Retro (n)	Mixed (n)	Age (yrs)	Duration (months)	Current pain VAS^a^
Whole group (*N* = 35)	6720 [5245, 8194]	3776 [3074, 4477]	3813 [3079, 4546]	20	1	14	18.8 ± 1.7	60 ± 33	4.8 ± 2.7
Less than 5 years. (*N* = 16)	5120 [3857, 6382]	2976 [2379, 3572]	2519 [1844,3193]	9	0	7	18.5 ± 1.5	36 ± 15	4.6 ± 3.9
More than 5 years. (*N* = 19)	8067 [5701, 10434]	4443 [3325, 5561]	4581 [3473, 5690]	11	1	7	19.0 ± 1.8	81 ± 30	6.0 ± 4.1

Data are shown as means ± standard deviations, unless stated otherwise

^a^ *VAS* Visual Analogue Scale (0–10)

### PFP symptom duration and current pain

**Fig. 2:**
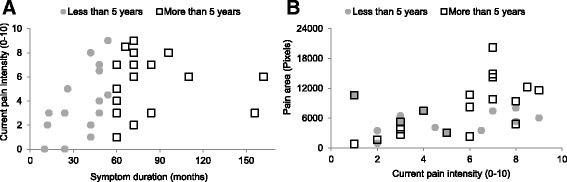
Positive correlations between symptom duration and current knee pain (**a**) and pain area and current knee pain (**b**) in adolescents and young adults with PFP symptoms. Correlations between pain intensity and symptom duration does not hold for those with symptom durations more than 5 years and conversely the correlation does not hold for pain area and current pain intensity for those with symptoms for less than 5 years

Based on the 5 year median split there was no difference in mean age (t(33), *p* = .339) or current pain (Mean difference −1.217, 95% CI [−3.088, 0.653]; t(33), *p* = .195), but a correlation between current knee pain and duration (r_s_ = 0.359, *p* = .034, Fig. 2) was found. Further analysis revealed that current pain intensity was correlated to symptom duration for those with pain for less than 5 years (r_s_ = 0.618, *p* = .011; Fig. 2a). An equivalent correlation could not be shown for individuals presenting with pain for more than 5 years (r_s_ = 0.193, *p* = .43; Fig. 2a).

### Supplemental files

Individuals PFP drawings included as a Additional file 1 (Individual PFP drawings.pdf) illustrate the details within the pain drawings and are presented according to the median split of ‘Symptom duration of less than 5 years’ page 1 and ‘Symptom duration of more than 5 years’ page 2.

### PFP area and current pain

For the 35 individuals with long-standing PFP, the total area of knee pain (sum of left and right knee pain area) was also found to be positively correlated to the current knee pain intensity (r_s_ = .465, *p* = .006), however further analysis revealed that this correlation only held for those with pain for 5 years or more (r_s_ = 0.621, *p* = .0045; Fig. 2b). The group of patients presenting with pain for less than 5 years showed no relationship between current knee pain intensity and area (r_s_ = .135, *p* = .637; Fig. 2b).

### PFP area and symptom duration

**Fig. 3:**
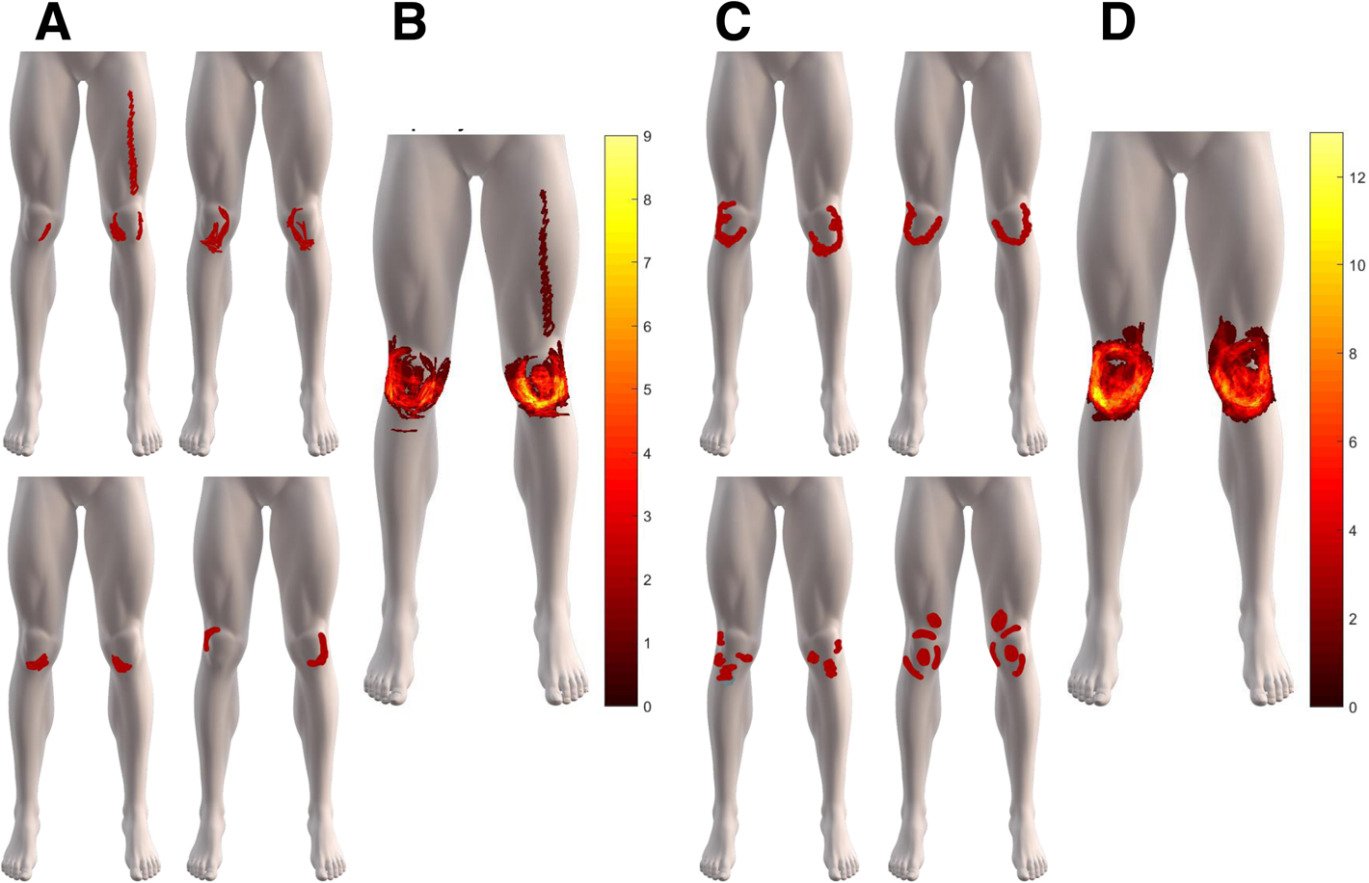
Representative patellofemoral pain drawings for **a** patients with symptoms for less than 5 years and **b** a corresponding overlay (*N* = 16). Representative patellofemoral pain drawings for **c** patients with symptoms for 5 years or more and **d** a corresponding overlay (*N* = 19)

Individuals presenting with knee pain for 5 years or more demonstrated a greater area of knee pain than those with pain for less than 5 years (Two-way Anova, F(1,58) = 11.523, *p* = .001; Table 1) with no differences in pain area between the left or right knee (Two-way Anova, F(1,58) = 0.0938, ns, Table 1). The greater area of knee pain, according to the median split, are visualized in Fig. 3 which depicts pain referral spreading towards the upper peripatellar region, converging to an ‘O’ shape, for those with ‘extended’ symptom durations of 5 years or more whilst those with pain for 5 years or less takes on a U shape.

### Symmetrical knee pain patterns in PFP

**Fig. 4:**
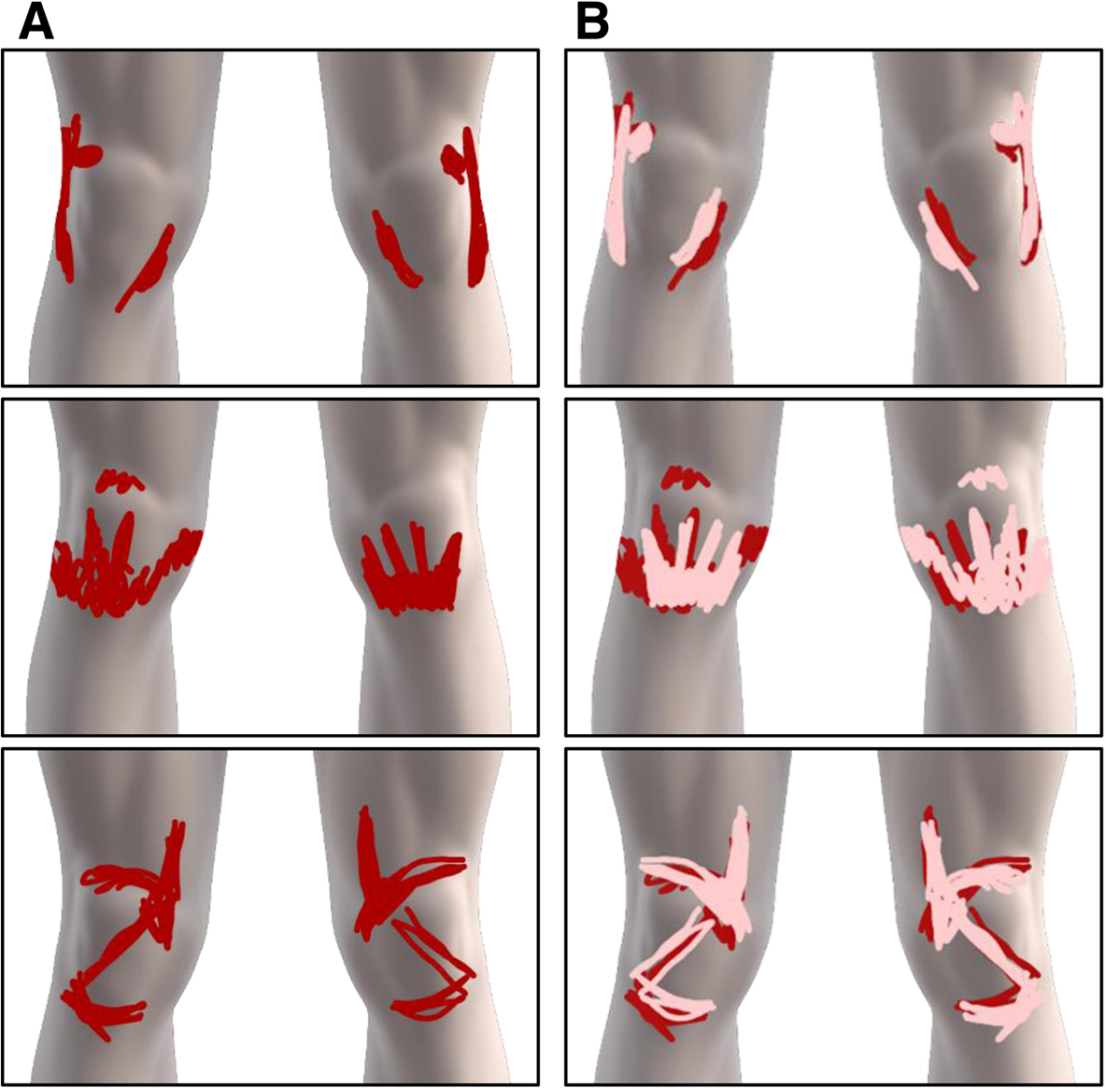
Representative expressions of three patients with bilateral PFP (**a**) demonstrating remarkably symmetric pain patterns drawn on the left and right knee. For the purpose of visualization, the mirrored/reflected image was superimposed onto the original (**b**) to create a comparison image

Of all knee pain patterns, 77% (27/35) of patients presented with pain in both knees and 82% (22/27) of those patients were classified as having symmetry between the pain pattern drawn on the left and right knee, refer to Fig. 4 for examples. In line with these results, the pain area between the left and right knee for those displaying symmetrical pain patterns did not differ (t(21), *p* = 0.729). The pain area associated with the worse side of knee pain did not differ from the contralateral knee for those presenting with bilateral or symmetrical knee pain patterns (Mean difference: 233 pixels, 95% CI [−138, 605]; t(21), *p* = .206).

### Objective assessments of symmetry in knee pain patterns

**Fig 5:**
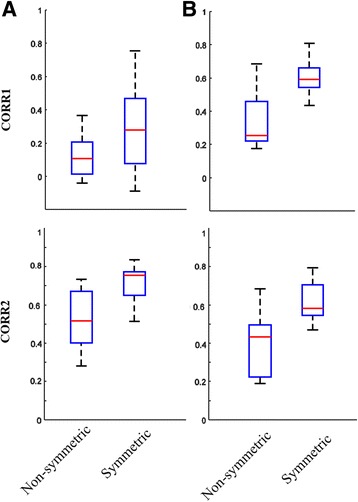
Classification of symmetry for the symmetry drawing task (SDT, **a**) and the PFP drawings (**b**) according to the PFP expert, the two-dimensional correlation coefficient (CORR1), and maximum correlation coefficient + Jaccard index divided by 2 (CORR2), showing that CORR1 alone is not able to discriminate between symmetry and non-symmetric pain drawings

The symmetry drawing task revealed that if persons intended to draw a mirrored or symmetrical knee pain pattern and were classified by an expert as being symmetric a CORR1 > = 0.5 could be expected, refer to Fig. 5. The patterns obtained from the symmetry drawing task that were classified as symmetric by the PFP expert but did not satisfy the CORR1 > = 0.5 threshold were translated and CORR2 computed in order to achieve a true positive rate (TPR) of 100%. This resulted in a simple fuzzy rule which states that symmetry in PFP drawings are mathematically satisfied if (CORR1 > = 0.5) OR (CORR2 > = 0.5). However, this rule resulted in a false positive rate (FPR) of 50% which is equivalent to four out of eight pain drawings. These results imply that the PFP expert is stricter at assigning symmetry in pain drawings from the symmetry drawing task.

Applying the simple fuzzy rule on all bilateral PFP patterns yielded a TPR of a 100% and false positive rate FPR of 25% for symmetrical PFP patterns. These results identify the expert PFP as more strict when assigning symmetry to a bilateral knee pain drawing obtained from PFP drawings.

## Discussion

This is the first study to illustrate and show the remarkably detailed expressions of knee pain patterns, as drawn on high-resolution 3D digital body-schema of the knees, from adolescents and young adults with long-standing PFP. Moreover, exploration and quantification of the knee pain area, location and distribution revealed PFP complaints were common in the lower peripatellar region and a somewhat specific expansion of pain spread tended towards the upper peripatellar region for those with an extended (greater than 5 years) duration of PFP symptoms. Only one individual with PFP presented with pain restricted to the retropatellar area while the majority presented with a pain pattern restricted to the peripatellar region. Furthermore, a high number of individuals with bilateral PFP presenting with a symmetrical pain pattern demonstrated a mirrored expression of pain in the left and right knee in terms of location, shape and size. Classification of symmetry in PFP was explored using subjective and objective mathematical approaches, which resulted in the finding that PFP experts may be stricter when visually classifying the degree of symmetry in PFP pain patterns.

### Pain localization

The pain localization found in the current study resembles previous work on adolescents with PFP and further highlights that the majority of individuals have peripatellar or peripatellar mixed with retropatellar pain [6]. In accordance to a separate study with young adults (mean age 21) the most common pain localization (83%) was in the peripatellar region [23]. Brushøj et al. and colleagues (2008) suggest that the report of pain within the peri-patellar region supports the notion that synovium is involved in the genesis of PFP. For this study, there is also clear spatial overlap for the majority, regardless of symptom duration, around the lower part of the patella which is consistent with proposed involvement of Hoffas fat pad in PFP [23].

The spatial overlap of the pain patterns is higher in the PFP group with extended pain duration and the pattern itself appears to progress from a “U” to an “O” shape around the peripaterllar region. The progression may reflect a generally less uniform or more variable pain pattern to a more uniform or less variable presentation of pain in a duration-dependent manner. If this is the case, the results give rise to the notion that there is a convergence towards a specific pattern, which is an “O” shape, as symptom duration progresses.

### Signature and symmetrical knee pain patterns

Sixty years ago, Palmer stated that symmetry “is almost diagnostic of a functional nervous disorder, or the functional superstructure which the patient may have built up around [an] organic lesion'' [24]. In this investigation, approximately three out of every four patients presented with bilateral PFP and the majority of those individuals expressed their on-going pain with a remarkable degree of similarity between the left and right knee. Of particular interest for this present study was the extraction of the common locations of pain, such that information about pain location with respect to retropatellar or peripatellar regions could be contrasted with known and underlying structures and pathophysiology of long-standing PFP. In consideration of earlier queries [25, 26] and pivotal investigations [27] on the topic of mirror-image pain and neurogenic inflammation, the emergence of symmetrical mirror-image bilateral ‘knee-pain’ patterns was difficult to interpret. To our knowledge, detailed reports of on-going symmetrical pain expressions within a complex structure, such as the knees in humans, have yet to be elaborated upon, or at best qualitatively reported. Indeed, the concept of symmetry in pain as a symptom, with regards to spatially-specific areas of symptom location and/or the degree of symptom progression as expressed on the left and right side of the body has been reported [28–30]. The symmetrical presentation of symptoms occurring in spatially specific areas is known to be a cardinal feature in diseases such as rheumatoid arthritis and psoriasis [26]; where symmetry is the norm rather than the exception. However, it has been proposed that patients presenting with asymmetrical pain may advance towards symmetry (symmetrization) as a disease (e.g. rheumatoid arthritis) progresses and therefore may be of importance for the clinical management of the disease [30]. To date, PFP is often described as diffuse anterior knee pain during activities that load the patellofemoral joint such as stair walking, walking or running and some may communicate their pain by ‘placing both hands over their knees to indicate the area of pain’ or ‘point to the area around the knee such that pain is described as taking a classic or signature *C-sign*’ [14]. Clearly, clinical recordings of referred pain area, location, and descriptions of the pain expression reported in association with PFP have received less attention in this respect. In related fields of knee pain, these features are however, within the top seven of the most important outcome variables for post-surgical knee pain success [31].

Mirror-image pain is traditionally known as pain experienced on both sides of the body, as a result of trauma or inflammation in one limb. The recognition that symptoms occur in symmetrical locations, such as within the left and right hand and ankle joints in rheumatoid arthritis, encouraged seminal and pioneering investigations on the contralateral effects of pro-inflammatory mediators induced by unilateral nerve injury [27, 32]. By the late 1990s the notion that unilateral peripheral nerve lesions affect spatially-specific contralateral (undamaged) structures gained momentum and it became clear that the contralateral effects of a peripheral nerve lesion were not limited to the up-regulation of pro-inflammatory mediators but that gene expression, physiologic and anatomical remodeling could also occur [25]. Even models of unilateral muscle overuse injuries have been shown to up-regulate the tachykinin system bilaterally [33]. Tendency towards symmetry as a disease progresses has been reported for patients with rheumatoid arthritis, such that the grade of damage becomes symmetrical between paired joints, as assessed by radiography [30]. Particularly relevant for this present study, is that unilateral joint inflammation of the cartilage can lead to bilateral degeneration of knee cartilage in rats [34] which supports the possibility that symmetrical mirror-image pain in long-standing PFP may be indicative of both mechanism and progression of PFP. A possibility for the emergence of symmetrical mirror-image knee pain in adolescents and young adults with PFP may simply be abnormal knee or joint biomechanics. Given that PFP is usually provoked by bilateral weight-bearing activities, such as stair climbing, walking or running, then abnormal knee or joint biomechanics may exacerbate or lead to the development of premature ‘overuse’ injuries in both knees. Thus, symmetrical pain or mirror-image PFP may simply reflect a bilateral exposure to ‘overuse’ of associated muscle and tissue knee structures. Nonetheless, it may be highly relevant to track the progression of PFP patterns and associated degree of symmetry as the disease or duration of symptoms progresses until a better understanding of the pathophysiology and driving mechanisms are clear.

In this study, we have explored and contrasted subjective and objective means of classifying bilateral PFP patterns as symmetric or non-symmetric. Further, we assessed the level of symmetry between two drawings that were intended to be symmetrical in order to threshold for natural pain drawing variation. Classical mathematical approaches for assessing symmetry could be ascertained with a 100% TPR and 20% FPR. Altogether the results showed that the PFP expert in this study was stricter when assigning symmetry classification as compared to the mathematical approaches. The FPR found in this study is attributed to the location differences in pain drawn on the left and right knee, with the PFP expert applying knowledge and weighting significance onto the underlying anatomical structures to guide symmetry classification. Nonetheless, the degree of symmetry in the PFP patterns are evident, quantifiable, and thought provoking.

It remains to be determined the time course of pain area expansion or spread in PFP symptoms as the condition progresses and whether (1) the pain area expansion is a key metric for symptom progression and (2) if symptoms progress towards symmetry with symptom duration and (3) if simple fuzzy rules and classification approaches then become more robust with symptom duration. Such data would be useful in determining if there is a systematic expansion in pain area as implicated by studies of knee pain drawings in knee osteoarthritis arthritis (OA) showing that global, rather than localized pain, is associated with worse self-reported pain and function [35].

A limitation of this study is the low number of subjects and the median split used to sub-divide the PFP symptom duration, and thus these results need to be replicated in a large external cohort of patients with PFP in order to get a better indication of the role. Recently it has been shown that the size of the pain drawing areas in knee OA patients was not specifically correlated to preoccupation, attention, catastrophizing, or fear of pain but rather indirect assessments of central sensitization [36]. Therefore, the presence and significance of the degree of symmetry, as revealed by pain drawings, should further explored as an objective measure of symptom progression.

### ‘Extended’ long-standing knee pain

In this study, a majority of those with long-standing PFP showed bilateral symmetrical mirror-image pain so subgrouping based on the presence or absence of this condition was not feasible (i.e. symmetrical vs. asymmetrical knee pain), and therefore currently limits further query in this regard. However, those with extended long-standing PFP indicated that the area of pain was greater than those presenting with PFP symptoms for less than 5 years. Moreover, those with PFP symptoms for 5 years or more showed a strong positive correlation between current pain intensity and area which supports theoretical models for development of chronic widespread pain [37]. Recent evidence suggest that adolescents with PFP demonstrate reduced pressure pain thresholds remote from the site of self-reported pain, which implicates altered central processing of sensory input [6]. Collectively, these findings challenge the current understanding and awareness of PFP in adolescents and young adults and supports that PFP should be regarded as “a knee condition with high rate of persistence and features of central involvement” [6, 9, 37].

## Conclusions

The use of high-resolution digital pain drawings enabled a detailed account of knee pain patterns and these patterns revealed variations in pain spread as well as underlying similarities that appear to emerge as symptom duration progresses. The high majority of those presenting with bilateral knee pain demonstrated symmetrical knee pain patterns should be further explored and gathering more information on how those with PFP symptoms initially report their pain may unravel the significance of this finding. Understanding what may be the natural progression of symptoms with time for those with PFP would be clinically valuable for both pain management and treatment. This present study provided a window into the possible pain patterns that may exist within PFP and provides rationale to further explore the significance of these knee pain patterns in larger cohorts.

## Additional file

Additional file 1: Individual PFP drawings. (PDF 828 kb)

## Abbreviations

2D: Two-dimenstional
3D: Three-dimensional
ANOVA: Analysis of variance
CI: Confidence interval
CORR1: Correlation coefficient
CORR2: Maximum correlation coefficient + Jaccard index
FPR: False positive rate
maxCORR: Maximum correlation coefficient
NRS: Numberical rating scale
PFP: Patellofemoral pain
SD: Standard deviation
S-pen: Stylus-pen
TPR: True positive rate
VAS: Visual analogue scale
