# Supplementary material for: Distribution and symmetrical patellofemoral pain patterns as revealed by high-resolution 3D body mapping: a cross-sectional study
Source: BMC Musculoskelet Disord. 2017 Apr 18;18:160. doi: 10.1186/s12891-017-1521-5 (PMC5395838; doi:10.1186/s12891-017-1521-5)

# Individual PFP drawings

***Symptom duration of less than 5 years.***

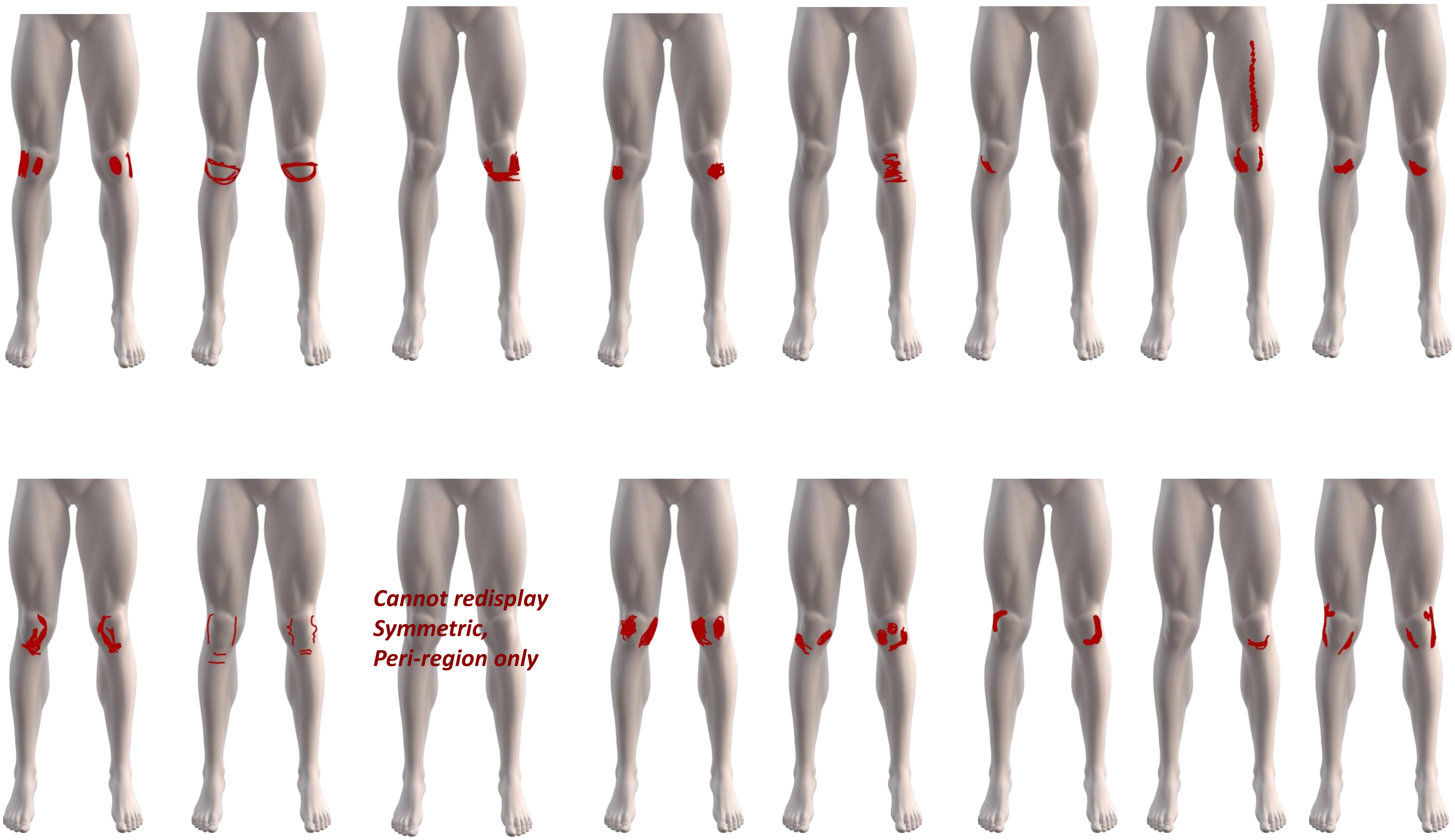

# Individual PFP drawings

*Symptom duration of more than 5 years.*

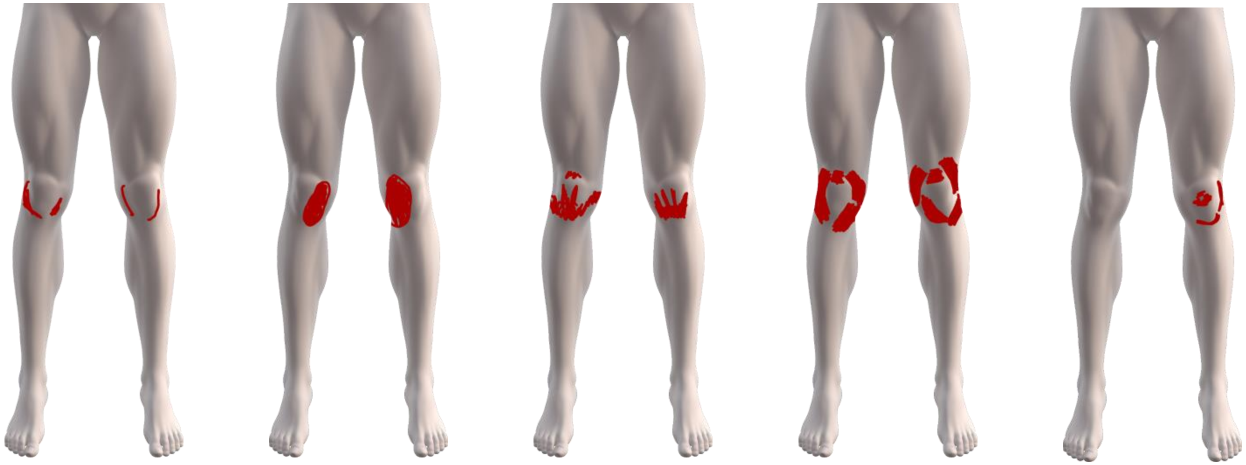

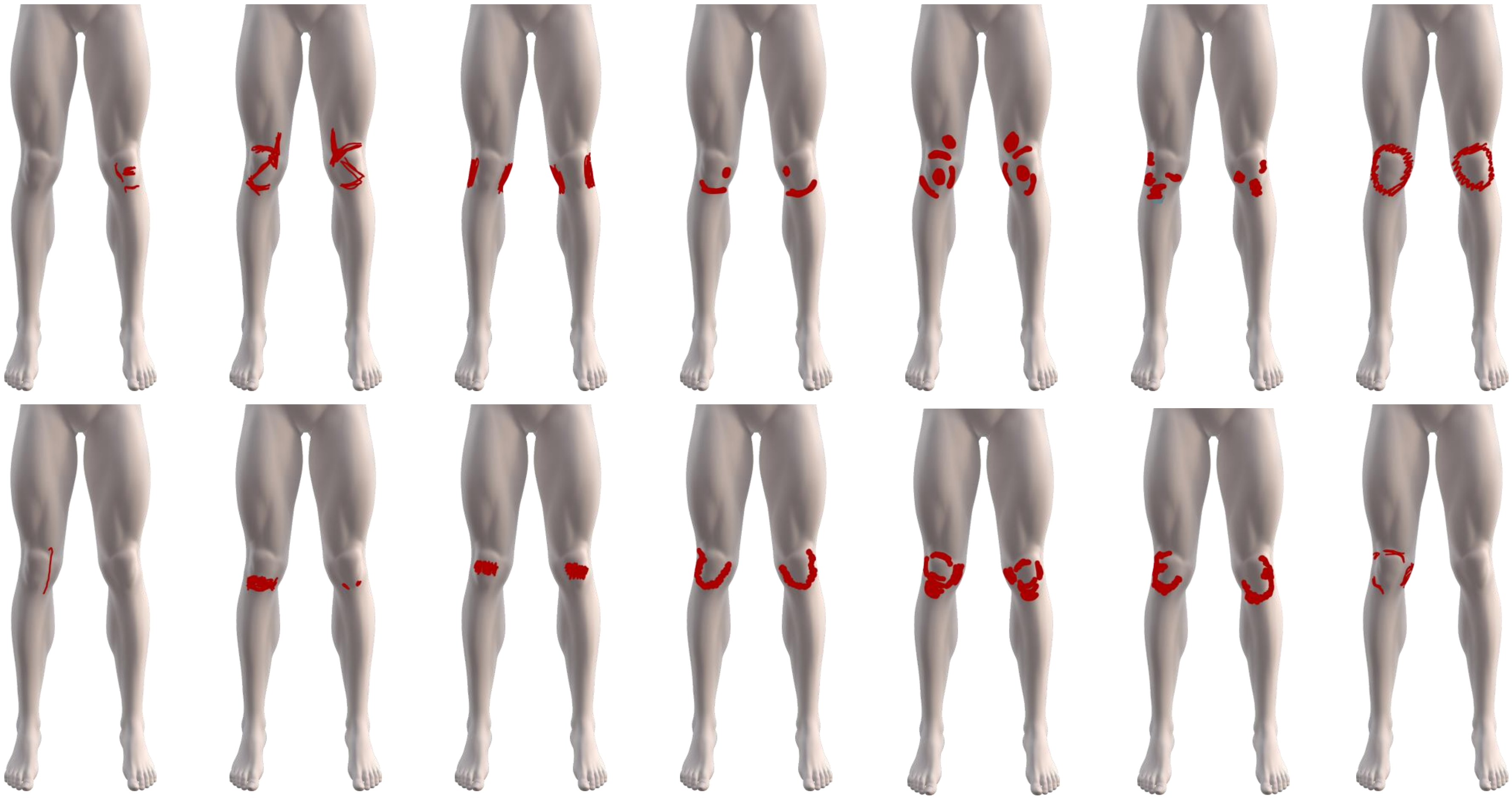

Supplement: Additional file 1: — Individual PFP drawings. (PDF 828 kb) [file 12891_2017_1521_MOESM1_ESM.pdf]
